# Long-Term Trends and Ecological Risks of Heavy Metal Accumulation in Cultivated Land of Songnen Plain, China

**DOI:** 10.3390/toxics13010059

**Published:** 2025-01-15

**Authors:** Zonglai Liu, Jinying Li, Yanan Chen, Fengjun Zhang, Wei Feng

**Affiliations:** 1 College of Jilin Emergency Management, Changchun Institute of Technology, Changchun 130012, China; liuzonglai@ccit.edu.cn (Z.L.); 20231311206@stu.ccit.edu.cn (J.L.); 2 College of Energy and Environment, Jilin University, Changchun 130026, China; zhangfengjun@jlu.edu.cn (F.Z.); weifeng@jlu.edu.cn (W.F.)

**Keywords:** agricultural soil, heavy metals, source apportionment, ecological risk, early warning

## Abstract

Heavy metal pollution in agricultural soils poses a serious threat to food security. Therefore, it is crucial to conduct risk assessments and issue early warnings about high levels of metal contamination for the sustained prosperity of agriculture. To assess the risks, identify the sources, quantify the amounts, and determine the extent of pollution from seven heavy metals, as well as to provide early warnings, 78 soil samples were collected from farmed lands in the Songnen Plain of Jilin Province. The average concentrations of Zn, Cu, Mn, Pb, Cd, Ni, and As were found to be 2.05, 1.5, 0.2, 1.09, 2.68, 1.53, and 1.17 times higher than the background values of Chinese soils, respectively. Source analysis indicated that toxic Pb originates from vehicle exhaust emission, while Cd, Cu, and Ni are attributed to industrial activities. Zn and As are likely associated with agricultural practices, and Mn predominantly stems from natural environmental sources. The geo-accumulation index suggests relatively high, accumulation levels for Zn, Cu, Mn, and Pb. Meanwhile, the single-factor pollution index indicates elevated pollution levels of Zn, Cu, and Cd. Potential ecological risk assessment reveals that certain areas within Changchun and Baicheng cities exhibit higher ecological risks. Notably, Cd has the highest potential ecological risk index (RI) of the seven heavy metals and warrants special attention. By analyzing annual pollutant accumulations, predictions can be made about the heavy metal content in four cities within the Songnen Plain, enabling the issuance of early warnings regarding soil heavy metal risks. The findings suggest that without proactive measures to mitigate heavy metal accumulation in soils, Changchun and Songyuan will reach severe pollution levels by 2031 and 2029, respectively.

## 1. Introduction

With the rapid development of industrialization and urbanization, global environmental issues are becoming increasingly severe [1,2]. Among these concerns, heavy metal contamination stands out as a major type of pollution due to its toxicity, persistence, non-biodegradability, and bioaccumulation [3,4,5]. Known for their high toxicity, heavy metals can linger in the environment for long periods. They accumulate in soil, sediments, plants, and animals, leading to harmful effects on both living and non-living components of ecosystems [6,7,8,9]. They can also enter the human body via the food chain, adversely affecting human health [10,11]. Agricultural ecosystems play a crucial role in supplying basic agricultural products to humanity [12]. It is estimated that 19.0% of China’s agricultural soils exceed established environmental quality standards for pollutants [13,14], indicating the severity of heavy metal pollution in China’s agricultural soils and its significant impact on soil quality. Therefore, exploring future trends of heavy metal accumulation in soil is critical for the prevention and control of such contamination.

Black soil, also known as Chernozem according to WRB classification, is renowned for its abundant organic matter, which binds with heavy metals to form complex compounds, thereby reducing the mobility of these metals effectively [15]. The Songnen Plain in Northeast China serves as a significant hub for both industry and agriculture, characterized by an extensive coverage of fertile black soil. According to the “2022 China Ecological Environment Bulletin” published by the Ministry of Ecology and Environment, heavy metals have been identified as the primary pollutants affecting the environmental quality of agricultural soil. Therefore, investigating heavy metal contamination in this region is of utmost importance.

Human activities, such as industrial emissions and agricultural practices, can directly or indirectly lead to the accumulation of heavy metals in topsoil [16]. Anthropogenic sources of heavy metals are transported through the air, accumulate on the soil surface via atmospheric deposition, and, subsequently, penetrate deeper into the soil profile [17]. Additionally, the presence of nearby industrial facilities or major roadways can contribute significantly to the accumulation of heavy metals in soil. Increased pollutant emissions Pb to heavy metals entering the atmosphere, where they eventually deposit into the soil [16,18]. Numerous methods are currently employed to evaluate the potential for excessive metal contamination in soil, including the Single Pollution Index (PI) [10,19], Nemerow Composite Pollution Index (PI_Nemerow_) [20,21], Geo-accumulation Index (*I_geo_*) [22], and Potential ecological risk (RI) assessment [23,24]. Among these, the geo-accumulation index is widely recognized as being particularly effective. Additionally, geostatistical and multivariate statistical models, such as principal component analysis, spatial autocorrelation analysis, and cluster analysis [25,26,27,28], are commonly used to distinguish the sources and spatial distribution characteristics of heavy metals.

To date, some scholars have attempted to use methods such as logistic regression [29], mass balance models [30], and machine learning [31,32] to predict the spatiotemporal changes in pollutants in soil. However, the performance of these methods varies significantly and is limited by data availability. Some researchers assess soil pollution by collecting samples over extended periods [33,34]. This method, however, is time-consuming and costly, and it cannot predict future pollution levels, making it less frequently used. Most soil pollution originates from human activities, particularly from industries that heavily exploit natural resources [35]. One effective approach to predicting future pollution and its associated environmental risks is to establish a clear correlation between pollution emissions from these sources and economic growth. This involves determining the rate at which pollutants accumulate [36]. To accurately forecast future pollution levels, it is essential to understand how pollution sources are evolving, their contributions, and the baseline levels of pollutants. Zhang and Li Zhang, Li [30] proposed a method for analyzing temporal changes in soil pollution levels. This method considers both the typical concentrations of contaminants and the severity of contamination hotspots. By using GDP as a proxy variable for pollution source activity intensity (AI), this study establishes a dynamic relationship model between economic activity and ecological risk. While studies have explored the link between GDP and pollution emissions, few have quantified the long-term impact of economic growth on ecological risks in conjunction with natural attenuation coefficients and pollutant accumulation processes. The novelty of this study lies in integrating economic activity index (GDP), pollution emission intensity (EI), and ecological risk index (RI) into a dynamic evaluation framework that transitions from economy to ecology. It examines the change trend of ecological risk under different economic growth scenarios (such as 6% and 3% growth rates), providing a reference for forecasting future development.

The main objectives from this research are as follows: (1) to assess the concentrations of heavy metals in agricultural soils of the Songnen Plain; (2) to analyze the sources of heavy metals within the Songnen Plain; (3) to evaluate the extent of heavy metal pollution and associated ecological risks in the soil; and (4) to provide early warnings regarding ecological risks posed by high levels of heavy metals in the soil. This study not only contributes to our understanding of heavy metal distribution and its environmental impacts but also provides critical data for developing targeted pollution control strategies. By identifying key sources of contamination and predicting ecological risks, this research supports informed decision-making for sustainable agricultural practices and environmental protection in the Songnen Plain.

## 2. Materials and Methods

### 2.1. Study Area

**Figure 1:**
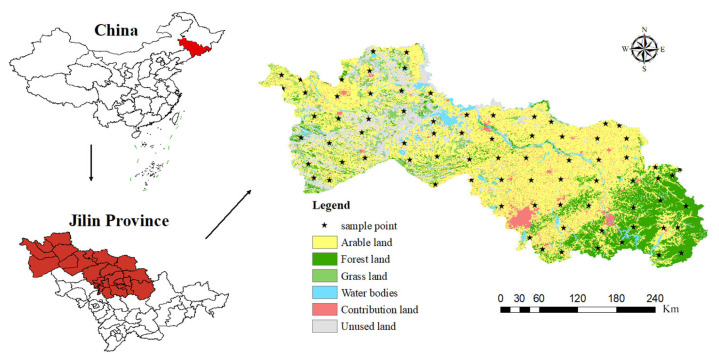
Map of the study area and sampling sites.

The Songnen Plain, named after the Songhua River and Nen River, is located within Heilongjiang and Jilin Provinces. This study focuses primarily on the portion of the Songnen Plain situated within Jilin Province (Figure 1). The plain is characterized by its flat terrain, with an average elevation ranging between 150 and 200 m. Long-term alluvial action from rivers such as the Songhua and Nen has formed a fertile alluvial plain, with soil types predominantly consisting of Phaeozems [37]—fertile soils that are highly suitable for agricultural production due to their high organic matter content and nutrient retention capacity. The region experiences a temperate continental monsoon climate, marked by an average annual rainfall of 600 to 700 mm, concentrated mainly in July and August. Annual evaporation is approximately 1620 mm, and the average annual temperature stands at 4.8 °C. The soils in the study area contain moderate levels of organic matter, supporting agricultural productivity.

### 2.2. Sample Collection and Analysis

In 2023, 78 composite soil samples were collected from the surface layer (0–20 cm) of the Songnen Plain, as depicted in Figure 1. Each composite sample comprised five individual soil samples gathered from a square plot measuring 1 km on each side (four samples from the corners and one from the center, each weighing 1 kg). Upon return to the laboratory, the soil samples were processed by removing debris, sieving through a 2 mm mesh, and air-drying at room temperature. They were then freeze-dried until they could be tested and were analyzed within six weeks. Given the extensive unit area of cultivated land and the similar farming practices across different regions of the Songnen Plain, these 78 composite samples provide a robust representation of heavy metal distribution over a large area.

The sample analysis was conducted with reference to Chinese national standard methods [38] and previous studies [39,40]. An amount of 0.5 g of soil from each sample was digested with HNO₃-H₂SO₄-HClO₄ in a 50 mL polytetrafluoroethylene crucible through heating, and the total contents of Zn, Cu, Mn, Pb, Cd, and Ni were determined using inductively coupled plasma mass spectrometry (ICP-MS, Avio, PerkinElmer Inc., Waltham, MA, USA). For As, another 0.5 g of the sample was digested with 10 mL of HNO₃-HCl in a 1:1 (*v*/*v*) ratio, and As was determined using atomic fluorescence spectrometry (AFS). Soil pH was measured with a pH electrode (Model PB-10, Sartorius, Gottingen, Germany) in a 1:2.5 (soil/distilled water) extract. The soil organic carbon (SOC) was determined using the titrimetric method [41].

### 2.3. Quality Assurance and Quality Control

We used national standard soil reference materials (GSS-21, GSS-25) to ensure quality control. The recovery rates for heavy metals and metalloids ranged from 87% to 112%. Quality assurance was further verified by testing each batch of samples with blank samples and duplicate samples. The relative differences between duplicate samples were less than 6.60% across all batch processes.

### 2.4. Heavy Metal Pollution Analysis

#### 2.4.1. Geo-Accumulation Indicator ($I_{geo}$)

The $I_{geo}$ is a sophisticated and specialized technique used to assess the impact of human activities on the environment and to measure the extent of soil pollution by accounting for natural variations in the distribution of heavy metals [42,43]. It achieves this by comparing the ratio of measured concentrations to background values in the soil environment. The calculation formula is as follows:

(1) $$I_{geo}=\log_{2}\frac{C_{n}}{1.5B_{n}}$$

Here, $I_{geo}$ represents the geo-accumulation index of heavy metal n, $C_{n}$ is the measured concentration of heavy metal n in the soil. $B_{n}$ is the geochemical background value of that heavy metal.

Based on the $I_{geo}$ values, pollution levels are categorized into seven grades, ranging from unpolluted to extremely heavily polluted. The seven grades proposed by Muller [44]) are as follows:

$I_{geo}\leq$0, uncontaminated

$0<I_{geo}\leq$1, uncontaminated to moderately contaminated

$1<I_{geo}\leq$2, moderately contaminated

$2<I_{geo}\leq$3, moderately to heavily contaminated

$3<I_{geo}\leq$4, heavily contaminated

$4<I_{geo}\leq$5, heavily to extremely contaminated

$I_{geo}>5$, extremely contaminated

#### 2.4.2. Single Pollution Index

The single pollution index method can quickly check how much heavy metal is building up in the soil [45,46,47]. The formula is as follows:

(2) $$P_{i}=\frac{C_{i}}{S_{i}}$$

where $C_{i}$ is the measured content determination of heavy metal I (mg/kg), $S_{i}$ is the environmental quality standard value for heavy metals in soil. Pollution levels can be divided into 5 categories: uncontaminated ($P_{i}\leq 0.5$), warning line ($0.5<P_{i}\leq 1$), light pollution ($1<P_{i}\leq 2$), moderate pollution ($2<P_{i}\leq 3$), severe pollution ($P_{i}>3$).

### 2.5. Ecological Risk Assessment of Heavy Metals

The Potential Ecological Risk Index (RI) [48] takes into account four factors: soil heavy metal concentration, pollutant type, toxicity level, and the sensitivity of the medium to heavy metal pollution. This method was proposed by Håkanson [49], and the formula is as follows:

(3) $$E_{r}^{i}=T_{r}^{i}\times P_{i}$$

(4) $$RI=\sum_{i=1}^{m}E_{r}^{i}$$

In the formula, $E_{r}^{i}$ represents the single-factor potential ecological risk index, RI denotes the overall potential ecological risk of pollution, $T_{r}^{i}$ is the toxicity factor. The toxicity factors for Zn, Cu, Mn, Pb, Cd, Ni and As are 1, 5, 1, 5, 30, 5, and 10, respectively. $P_{i}$ is single factor index. The evaluation criteria for $E_{r}^{i}$ can be categorised as low risk ($E_{r}^{i}$ < 40), medium risk (40 ≤$\;E_{r}^{i}$ < 80), considerable risk (80 ≤ $E_{r}^{i}$ < 160), high risk (160 ≤ $E_{r}^{i}$ < 320) and extremely high risk ($E_{r}^{i}$ ≥ 320). The ecological risk rating of RI was categorized as low risk (RI < 70), medium risk (70 ≤RI< 140), very high risk (140 ≤ RI < 280) and extremely high risk (RI ≥ 280).

### 2.6. Temporal Dynamic Trends of Heavy Metals in Soil

#### 2.6.1. Dynamic Source Release Modeling

When pollution occurs, hazardous materials naturally migrate from the affected locations into the surrounding soil and gradually spread to other areas [50]. To investigate this process, it is essential to examine EI, which refers to the amount of pollution originating from a source. EI depends on the total pollution produced by that source over time [51]. It can be calculated by summing the AI of the source over time, incorporating a decay factor [52]. There is a clear connection between EI and AI [53]. According to this study, pollution originates from all manufacturing operations in the region. To understand how pollution from a source changes over time, we need to analyze yearly data for that pollution source. These data are linked to the country’s economic growth (GDP) [54]. The formula is as follows:

(5) $$EI_{m}\propto \sum_{2002}^{m}AI_{m}\times K^{m-2002}$$

where $EI_{m}$ and $AI_{m}$ denote the source EI and AI for m (m = 2002, 2003, …, 2035) years, respectively. *K* is the natural degradation rate of pollution (K = 0.95).

The AI data from 2002 to 2022 focused on the GDP of Changchun City [55], as GDP growth in other cities within the Songnen Plain did not exhibit significant changes. To illustrate how pollution trends evolve over time, the GDP of Changchun City was used as a proxy for the overall economic activity of the Songnen Plain. Each year’s total economic output was adjusted by dividing it by the Consumer Price Index (CPI) to account for inflationary effects. We conducted simulations to estimate AI values for the years 2023 to 2035 under three scenarios: (a) extrapolating future trends based on historical data; (b) assuming an annual GDP growth rate of 6% starting from 2023; and (c) assuming an annual GDP growth rate of 3% starting from 2023.

#### 2.6.2. Temporal Dynamic Trends in Heavy Metal Accumulation

The annual amount of pollution from various sources that accumulates in the soil is related to EI, which helps quantify the yearly pollution contributions from those sources. This formula can be used to forecast the temporal distribution of contaminants.

(6) Ām, i=Ā2022, i×(∑2022mEImEI2022)×Km−2022

${Ā}_{m,\;i}$ is the average amount of pollutant i in the soil of a town over m years.

### 2.7. Statistical Analysis

We conducted a Pearson correlation analysis using SPSS version 26.0 to examine the relationships between heavy metals found in the soil. The R programming language, version 4.2.2, was utilized to compile data related to industrial processes that contribute to heavy metal contamination in soil. Principal Component Analysis (PCA) with varimax rotation was performed using SPSS version 26.0 to simplify and identify the key sources of heavy metals. The spatial distribution of heavy metal pollution in the soil was visualized on a map using ordinary kriging interpolation methods in ArcGIS 10.7.

## 3. Results and Discussion

### 3.1. Descriptive Statistics of Heavy Metal Content in Soil

Table 1 presents data on heavy metal concentrations within the research area, where median values for Pb, Zn, and Mn significantly deviate from maximum values and are considerably higher than minimum values. This discrepancy likely reflects the substantial influence of specific pollution sources such as industrial emissions or agricultural activities in certain localized areas, indicating significant point-source contamination. Notably, the maximum values of Zn (exceeding its background value of 74.2 mg/kg) and Mn (exceeding its background value of 583 mg/kg) suggest considerable contamination from exogenous inputs, necessitating an in-depth investigation into the precise sources and pathways of these pollutants. Similarly, the maximum values of Pb and Cd also surpass their respective background levels, likely due to industrial or agricultural activities. Of particular concern is that the maximum values of Zn and Pb exceed the Risk Screening Values (RSVs) of 200 mg/kg and 140 mg/kg, respectively, implying potential risks to soil ecosystems and agricultural product safety in certain regions. The maximum value for Cd exceeded its RSV of 0.6 mg/kg, with a coefficient of variation (CV) as high as 0.63. This highlights the possibility of local hotspots where Cd concentrations may exceed the RSV value and require focused attention. The higher coefficients of variation for Zn, Mn, Pb, and Cd indicate greater sensitivity to industrial activities, whereas the lower coefficients of variation for Cu, Ni, and As suggest that these metals are less influenced by anthropogenic factors, possibly due to their predominant natural origins or differing biogeochemical behaviors. Such insights into the differential behavior of heavy metals enhance our understanding of pollution dynamics and support the development of targeted pollution control strategies to ensure soil quality and agricultural product safety.

**Figure 2:**
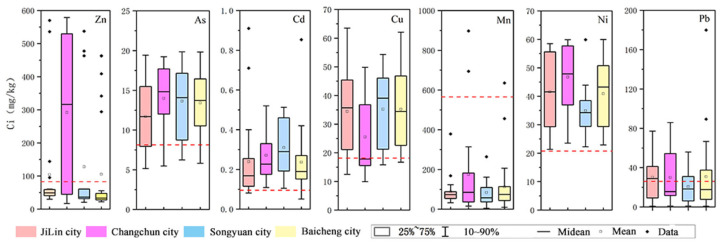
Box plots of the concentrations of seven heavy metals in agricultural soil samples from four cities in the Songnen Plain.

Figure 2 presents integrated data on heavy metal concentrations in agricultural soils across four cities in the Songnen Plain. The red dashed line in Figure 2 represents the background values of soil elements in the study area (Jilin Province). The results indicate that, except for Mn, the concentrations of all other heavy metals exceed the background levels to varying degrees [56]. Among these cities, Zn concentration is the highest in Changchun (with a maximum value approaching 600 mg/kg), significantly exceeding the background value. Research suggests that the application of fertilizers in agricultural soils contributes to the accumulation of Zn and Cd in the soil [57]. The concentration range of As is similar across all cities, with a concentrated distribution, indicating minimal spatial variation in As pollution. The concentration range of Cd is relatively higher in Songyuan, exceeding the background value, whereas Jilin has the lowest concentration, suggesting a lower risk of Cd pollution in this region. The concentration range of Cu is relatively consistent across the four cities, but the ranges in Songyuan and Baicheng are slightly wider, indicating potential localized pollution sources. The Ni concentration range in Jilin is relatively wide, with the maximum value approaching 60 mg/kg. The distribution of Pb is very similar across the four cities, with some concentrations exceeding the background value, indicating a certain level of Pb risk in all four cities. Overall, there are significant spatial differences in soil heavy metals in the Songnen Plain, and Figure 2 visually demonstrates these differences, which will aid in identifying high-risk areas and provide scientific support for soil pollution management and risk assessment.

We integrate specific findings from Table 2 into a cohesive discussion. Compared with previously reported heavy metal concentrations in agricultural soils, the mean concentration of Zn in our study area is higher than that observed in Chile (148.4 mg/kg), Ireland (72.5 mg/kg), America (66.4 mg/kg), and Cuba (90.7 mg/kg). This suggests potentially more intensive anthropogenic activities such as industrial emissions or extensive use of fertilizers, which are known contributors to elevated Zn levels. The mean Cu concentration exceeds levels found in Ireland (19.2 mg/kg) and America (24.9 mg/kg) but remains lower than those in India (48.7 mg/kg), Cuba (83.7 mg/kg), and Chile (669.4 mg/kg). This indicates moderate Cu pollution possibly linked to agricultural practices, while still being within globally varied ranges. Mn concentrations are notably lower compared to those in India (165.7 mg/kg) and Chile (551 mg/kg), reflecting regional differences in soil composition and possibly lesser impacts from external sources. The mean Pb concentration surpasses levels recorded in Italy (18.9 mg/kg), Ireland (20 mg/kg), America (26.5 mg/kg), and India (15.7 mg/kg), but remains lower than those in Chile (62 mg/kg), pointing to significant Pb contamination likely stemming from historical industrial activities or improper waste management practices. For Cd, the mean concentration is higher than in Ireland (0.2 mg/kg) but lower than in Italy (0.6 mg/kg), America (0.32 mg/kg), India (1.6 mg/kg), Chile (0.9 mg/kg), and Cuba (1.2 mg/kg). This indicates a moderate risk level for this highly toxic element, which can have severe implications for both environmental and human health. The average Ni concentration is greater than in Chile (15.6 mg/kg), America (23.6 mg/kg), and Ireland (12.5 mg/kg) yet lower than in India (92.9 mg/kg) and Cuba (294.2 mg/kg). This suggests varying degrees of Ni pollution influenced by regional industrial and agricultural practices. As concentrations exceed those documented in Ireland (3.2 mg/kg) and Cuba (10.8 mg/kg), highlighting a potential concern given As’s toxicity and its adverse effects on ecosystems and human health. The comparison demonstrates that the characteristics of heavy metal pollution vary under different backgrounds of human activities.

**Table 1:** Descriptive statistics of heavy metal concentrations in agricultural soils of Songnen Plain (mg/kg).

Parameter	Minimum	Maximum	Median	Mean	SD	CV	BV ^a^	RSV ^b^
Zn	17	579	46	152.47	191.56	1.26	74.2	200
Cu	9.93	63.5	33.83	32.79	14.05	0.43	22.6	200
Mn	4.72	897.4	73.75	115.22	150.64	1.31	583	—
Pb	0.71	179.83	18.33	28.46	28.64	1.01	26	140
Cd	0.051	0.91	0.197	0.26	0.16	0.63	0.097	0.6
Ni	21.35	59.95	38.49	41.27	12.32	0.30	26.9	100
As	5.15	19.85	13.73	13.15	4.28	0.33	11.2	25

SD: standard deviation; CV: coefficient of variation. a: soil element background values in China. China Environmental Science Press, Beijing, China [58]; b: Soil environmental quality—risk control standard for soil contamination of agriculture land. GB15618-2018 [59].

**Table 2:** Comparison of heavy metal concentrations with those in other countries and regions reported in previous literature.

Location	Average Concentration (mg/kg)							Reference
	Zn	Cu	Mn	Pb	Cd	Ni	As	
America	66.4	24.9		26.5	0.32	23.6		[60]
India	76.2	48.7	165.7	15.7	1.6	92.9	14.8	[2]
Chile	148.4	669.4	551	62	0.9	15.6	38.1	[34]
Ireland	72.5	19.2		20	0.2	12.5	3.2	[61]
Cuba	90.7	83.7		34.6	1.2	294.2	10.8	[62]
Italy				18.9	0.6		13.4	[63]

### 3.2. Source Analysis

**Figure 3:**
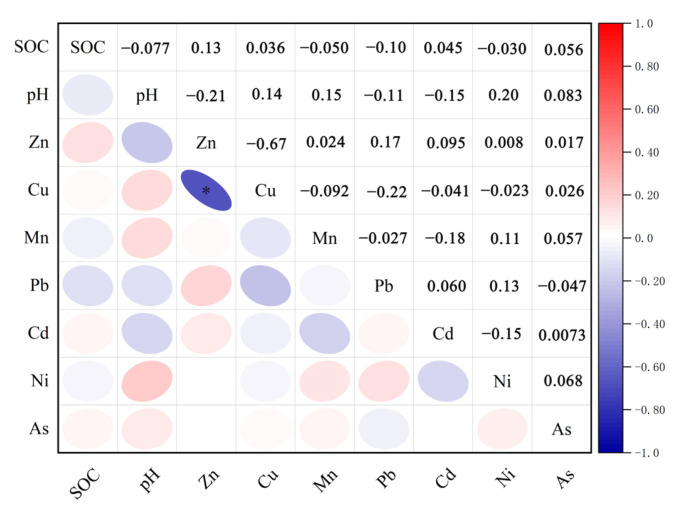
Pearson correlation analysis of heavy metals with soil pH and organic carbon. * The significance level is less than or equal to 0.01.

Heavy metals in the soil are interconnected due to their sources, mobility, and transformation processes [64]. In this study, Pearson correlation analysis, PCA, and cluster analysis were employed to identify the sources of heavy metals in the soil. The results of the Pearson correlation analysis for heavy metals are depicted in Figure 3. Zn showed positive correlations with all heavy metals except Cu. At a significance level of 0.001, significant positive correlations were observed between Cu and As, Mn and Ni, Mn and As, Pb and Cd, Pb and Ni, Cd and As, and Ni and As. Conversely, Cu exhibited negative correlations with all heavy metals except As, with significant negative correlations at the 0.001 level noted between Mn and Pb, Mn and Cd, Pb and As, and Cd and Ni. Negatively correlated heavy metals suggest different sources, while positively correlated metals may originate from similar industrial activities [65]. Therefore, Zn, Mn, Pb, and Cd might stem from the same industrial activities, whereas Zn and Cu are unlikely to be released simultaneously. Data on pH and SOC are presented in Table 3. Soil pH measurements in the study area ranged from 5.19 to 8.25, with a mean of 6.93 and a standard deviation of 0.8, indicating that the soil is generally neutral with little variation in acidity and alkalinity. SOC content ranged from 15.05 g/kg to 22.99 g/kg, averaging 18.92 g/kg with a standard deviation of 2.46, indicating fluctuations among different samples. Additionally, pH was negatively correlated with Zn, Pb, and Cd but did not show significant positive correlations with other heavy metals. Soil pH can influence the bioavailability of heavy metals, leading to their accumulation [66]. SOC was also correlated with Zn, Cu, Cd, and As. The accumulation of heavy metals can adversely affect soil microorganisms, limiting the degradation of organic matter and promoting the accumulation of SOC [67].

We used PCA to examine the heavy metals in the soil and identify the primary components accounting for the observed variations, resulting in four principal components with a cumulative variance of 73.4% (Table 4). PC1 explains 24.8% of the total variance, with Zn as the primary loading variable. Zn concentrations exceed background levels, suggesting human activity influences this element. Studies indicate that long-term use of Zn-containing fertilizers in agriculture can elevate soil Zn levels [68,69]. Cu has a significantly negative loading in PC1, indicating a different source from Zn, consistent with the previous correlation analysis. Cu is commonly used in industrial processes such as metal processing, wire and cable manufacturing, and electrical product fabrication. Therefore, PC1 reflects a combination of industrial and agricultural pollution. PC2 accounts for 17.782% of the variance, with Cd as the primary loading factor. Cd in agricultural soils often originates from phosphorus fertilizers, especially those with low phosphorus content. Given the developed automotive industry in Changchun, Cd poses a higher risk in the study area. Mn shows a significantly negative value in PC2, with relatively high background levels, indicating natural Mn presence. Thus, Mn is considered primarily of natural origin, and PC2 represents industrial pollution. PC3 explains 16.051% of the variance, with Pb and Ni as primary loading variables. Tire wear, gasoline additives, and motor vehicle exhaust emissions are main sources of Pb [70]. Ni is widely used in electroplating, metallurgy, battery manufacturing, and other metal processing industries. Therefore, PC3 is hypothesized to represent traffic and industrial pollution sources. PC4 accounts for 14.751% of the variance, with As as the key factor. Studies have shown that using fertilizers and pesticides can increase Cu and As levels in soil [71,72]. Hence, PC4 represents agricultural activities.

**Figure 4:**
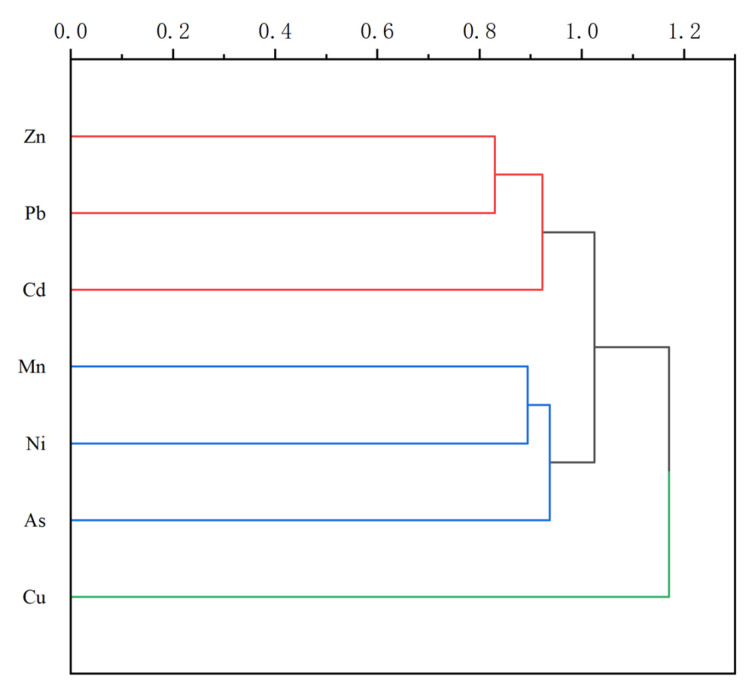
Cluster analysis of seven heavy metals.

To validate the PCA results, we conducted a cluster analysis on the seven heavy metals (Figure 4). Zn, Pb, and Cd formed a strong relationship and clustered with Mn, Ni, and As. The interaction between Zn and Pb may be linked to the adsorption of divalent metal ions such as Pb²⁺ and Zn²⁺ by aluminum hydroxide colloids [73]. Phosphate fertilizers contain significant amounts of Cd, contributing to its presence in farmland soils [74]. Unlike other heavy metals, Cu formed an independent cluster, showing negative correlations with Zn and Cd, indicating a different source. The common use of Cu in industrial processes suggests that it likely originates from industrial emissions. The Songnen Plain, a crucial agricultural region in China with extensive farmland and a long farming history, demonstrates that agricultural activities significantly impact heavy metal levels in the soil. Overall, the cluster analysis supports the findings of the principal component analysis.

### 3.3. Heavy Metal Pollution and Risk Evaluation

#### 3.3.1. Analysis of $I_{geo}$

The $I_{geo}$ was used to assess the accumulation of heavy metals in the agricultural soils of the Songnen Plain, and the background values of soil elements in Jilin Province were used as reference standards.

**Figure 5:**
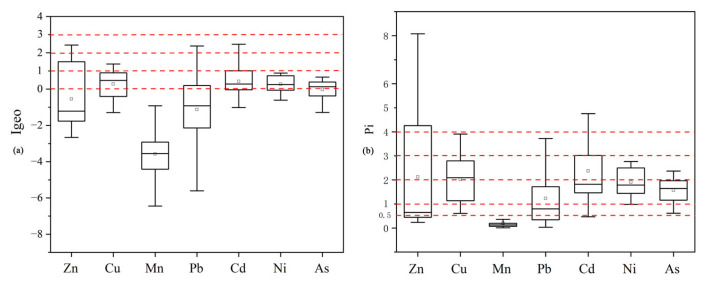
Geological cumulative index box plots (**a**) and single factor index box plot (**b**).

In agricultural soils of the Songnen Plain, the absolute values and allowable levels of Cd, Ni, and As are relatively low (Figure 5a), whereas the concentrations of Zn, Cu, Mn, and Pb are notably higher. The $I_{geo}$ revealed that seven sampling sites exhibited moderate Zn contamination, with three located in Baicheng City and four in Changchun City, accounting for 9% of all tested sites. Additionally, one site in Jilin City transitioned from being uncontaminated to moderately contaminated, representing 1% of the total number of sites tested. Ten sampling sites showed medium-level Cu contamination: four in Baicheng, one in Songyuan, four in Jilin, and one in Changchun, comprising 12.8% of the total number of sites. A total of 39 sampling sites, primarily in Baicheng and Songyuan, accounted for 50% of the total number of sites. Seventeen sampling sites exhibited medium-level Cd contamination, mainly concentrated in Baicheng, representing 21.8% of the total number of sites. Furthermore, 35 sampling sites transitioned from uncontaminated to moderately contaminated, accounting for 44.8% of the total. Notably, no sites showed moderate contamination for Ni and As. In conclusion, the accumulation of Zn, Cu, and Cd in the soils of the Songnen Plain should be a cause for concern for policymakers.

#### 3.3.2. Analysis of Single Pollution Index

The average order of the $P_{i}\;$ values for heavy metals in the agricultural soils of the Songnen Plain is as follows: Cd (2.37) > Zn (2.13) > Cu (2.02) > Ni (1.90) > As (1.57) > Pb (1.23) > Mn (0.20). Among these, Cd has the highest average $P_{i}$ value of 2.37, and 97.4% of the samples were above the warning level. The levels of pollution are divided as follows: 44.9% is mild, 20.5% is moderate, and 25.65% is severe. The average level of Zn is the second highest after Cd, with 62.8% of the samples going over the warning limit. The amounts of mild, moderate, and severe pollution are 1%, 1%, and 27%, respectively. Also, the $P_{i}$ values for Cu at all the testing locations are higher than the warning level. The amounts of pollution are classified as mild (32%), moderate (42.3%), and severe (12.8%) (see Figure 5b).

Ni and As have 55% and 59% of the sampling points, respectively, evaluated as mildly polluted. The $P_{i\;}$ value for Mn is very low, with 96% of the sampling points classified as unpolluted. Overall, Cd, Zn, and Cu in the study area show varying degrees of pollution, and these three heavy metals pose greater risks compared to the others. Studies have found that prolonged exposure to Cd can have various effects on the human body, including cancer, diabetes, cardiovascular diseases, and kidney damage [75], Additionally, excess Zn can also cause damage to the human nervous system [76]. Among the seven heavy metals examined, Mn poses the least risk.

#### 3.3.3. Single-Factor Potential Ecological Risk Index

**Figure 6:**
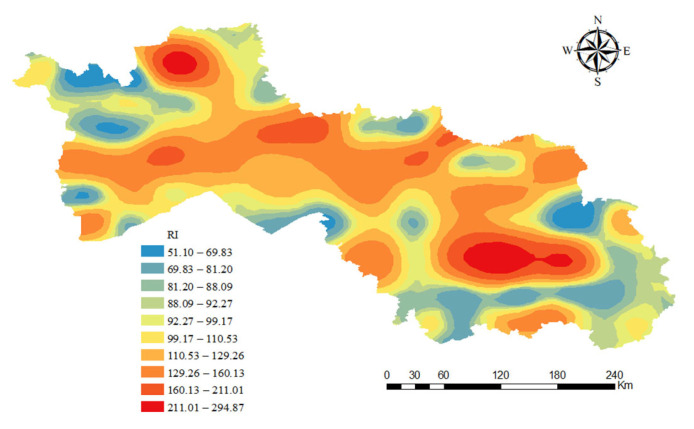
Distribution of potential ecological risks.

Based on the analysis of heavy metal contents in soil samples, the ecological risk of each heavy metal was assessed using a single-factor potential ecological risk index ($E_{r}^{i}$). The results showed that the potential ecological risks of most heavy metals belonged to the low-risk category ($E_{r}^{i}$ < 40), including Zn, Cu, Mn, and Pb, with maximum $E_{r}^{i}$ values of 8.08, 19.55, 1.56, and 38.96, respectively; however, Cd had a maximum $E_{r}^{i}$ value of 249.77, which belonged to the “high risk” category (160 ≤ $E_{r}^{i}$ < 320), so the potential ecological risk of Cd to the soil in this area is more significant. In addition, the maximum value of the composite RI was 294.87, which was in the “extremely high risk” range (*RI* >280). This result indicates that although most of the heavy metals have a low impact on the soil, the high risk level of Cd significantly increases the overall ecological risk of the soil in this area (Figure 6, Table 5).

Specifically, Cd poses the greatest risk, with the highest ecological hazard among all the heavy metals. The ecological risks of the other heavy metals are relatively small. Cd contributes an average of 61.89% to the *RI*, while the remaining six heavy metals contribute only 38.11% on average. The $E_{r}^{i}$ values for Cd range from 14 to 249.77, with 3.8% of sampling points classified as high risk, 27% as considerable risk, 41% as moderate risk, and 18% as low risk, indicating an overall higher risk level. Most of the sampling points are located in agricultural soil, industrial areas, and near highways, where these factors contribute to the increased levels of heavy metals in the soil and higher risks. Therefore, it is essential to strengthen the regulation of industrial enterprises and to use pesticides and fertilizers rationally in agricultural activities to effectively reduce pollution.

The places with higher levels of risk from soil heavy metals are mostly in the northwestern parts of Changchun City and Baicheng City (Figure 6). The contribution rates of Zn, Cu, Mn, Pb, Ni, and As to the total potential ecological risk are1.86%, 8.78%, 0.17%, 5.37%, 8.27%, and 13.66%, respectively. Sufficient attention should be given to the ecological risk caused by Cd, as it has the highest contribution rate to the overall risk.

### 3.4. Early Warning of Heavy Metal Risks

**Figure 7:**
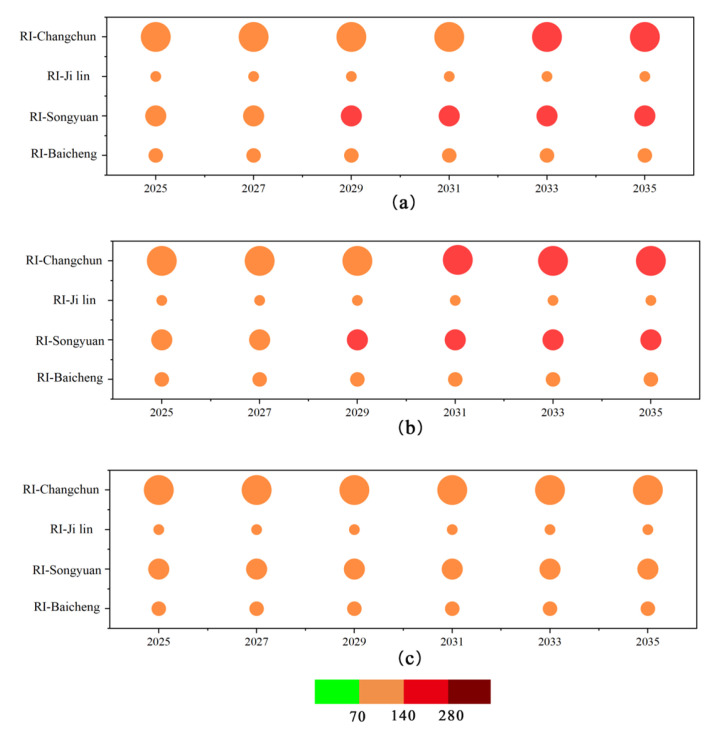
Projections of potential ecological risk indices for heavy metals in soils from 2025 to 2035 (**a**) based on fitted analysis and projections; (**b**) assuming a gross domestic product (GDP) growth rate of 6 per cent per annum; (**c**) assuming a GDP growth rate of 3 per cent per annum.

Figure 7 illustrates the projected future ecological risk of heavy metals based on predicted average annual pollutant levels, with three distinct scenarios modeled starting from 2023. The highest risk to soils is projected under a scenario assuming a 6% annual GDP growth rate. The second-highest risk is estimated using a fitted and projected trend of GDP over the past 20 years. A lower risk is anticipated when the annual GDP growth rate is 3%. To highlight variations in RI values, Figure 7 presents data for selected years: 2025, 2027, 2029, 2031, 2033, and 2035.

According to the classification standard for RI values, the index is divided into four levels. Under Scenario b, Changchun City and Songyuan City are each projected to escalate by one level, reaching the severe pollution level in 2031 and 2029, respectively. In Scenarios a, b, and c, Jilin City and Baicheng City are expected to maintain a moderate pollution level over the next decade. Notably, under Scenario a, the ecological risk in Changchun City is forecasted to reach the severe pollution level by 2033. Across all three scenarios, Jilin City and Baicheng City will remain at a moderate risk level over the next ten years. Overall, the ecological risk in the Songnen Plain is anticipated to remain at a moderate risk level over the next decade. In fact, due to their non-degradable nature, heavy metals will continue to accumulate in the soil unless measures are taken to restrict emissions from pollution sources. Evaluating the soil health risks across these scenarios reveals that managing GDP growth alone is insufficient to significantly reduce ecological risks. Even with an annual economic growth rate of 3%, environmental issues in the soils of cities within the Songnen Plain remain serious. While it is not feasible to halt all industrial activities, reducing pollution through better fuel choices, adopting new technologies, and modifying industrial practices can help mitigate the accumulation of heavy metals in the soil. This approach is crucial for minimizing future risks to the soil environment.

One limitation of this study is the use of GDP as a proxy for pollution source. While GDP effectively reflects economic activity levels, it does not account for factors such as technological advancements, policy measures, or shifts in industrial structure that can influence pollution emissions. Future studies could refine the model by integrating additional indicators, such as sectoral economic data, industrial emissions, or regulatory policies, to capture the complexity of the relationship between economic growth and ecological risk. Moreover, the relationship between GDP and ecological risk is not direct. Ecological risk is influenced by the accumulation and toxicity of pollutants, which are determined by EI and environmental factors. Therefore, GDP serves as a preliminary indicator for long-term trends, rather than a definitive measure of risk.

### 3.5. Uncertainty Analysis

There might be some mistakes in how this study predicts soil risk. This is mainly for two reasons: (1) Using Changchun City’s GDP to represent the whole Songnen Plain overlooks the growth of other cities, which could make the predictions seem higher than they really are. (2) Because of changes in industry and technology, the way GDP and yearly pollution levels relate to each other in the future might be different than it used to be. This factor could also make the predicted soil ecological risk values seem higher than they really are. Even with these unknowns, the overall results still show a careful pattern based on chemical risk levels. Also, outside things like temperature, rainfall, and closeness to rivers can affect how heavy metals move in the soil. This can cause the levels of heavy metals in the same area to go up or down in the future, which may lead to different warning results.

## 4. Conclusions

In this study, an analysis of 78 soil samples from the Songnen Plain revealed that maximum concentrations of Zn and Mn were significantly higher than background values, indicating substantial exogenous contamination. Pb and Cd levels also exceeded their respective background values, with Zn and Pb surpassing the RSVs for agricultural land. This could pose potential risks to soil ecosystems and agricultural safety. Further investigation showed that Changchun City exhibited the highest Zn concentration, reaching nearly 600 mg/kg, which is substantially above the background level. This elevated concentration may be attributed to agricultural fertilization practices. The study found that Mn primarily originates from natural sources, while Zn and As are mainly associated with agricultural activities. Pb is predominantly sourced from motor vehicle emissions, Cd from phosphate fertilizers used in agriculture, and Cu and Ni from industrial emissions. Our findings indicated that Zn, Cu, and Cd were the most severely polluted elements, with Baicheng City and Changchun City facing the highest risks of increasing soil heavy metal concentrations. Single factor pollution index results highlighted that Pb, Zn, and Cu exhibited the most severe pollution levels. Among all heavy metals, Cd posed the highest ecological risk and warrants particular attention.

By forecasting pollution source activity and examining annual contaminant accumulation, we predicted future soil ecological risks in the Songnen Plain. We considered three scenarios: (a) fitting and analyzing historical data to project future trends; (b) a 6% annual GDP growth rate starting from 2023; and (c) a 3% annual GDP growth rate starting from 2023. If adequate environmental protection measures are not implemented, Changchun and Songyuan Cities are projected to reach severe pollution levels by 2035. Efforts should focus on enhancing industrial production processes, restricting the use of polluting materials, and minimizing pesticide and fertilizer applications in agriculture to mitigate ecological risks. This research is essential for early risk warnings and the prevention and control of agricultural soil pollution.

## Figures and Tables

**Table 3:** Description of soil pH and SOC conditions.

	Max	Min	Average	Standard Deviation
pH	8.25	5.19	6.93	0.8
SOC (g/kg)	22.99	15.05	18.92	2.46

**Table 4:** Compositional matrix after rotation of each trace heavy metal loading.

	Principal Component Load			
	PC1	PC2	PC3	PC4
Zn	0.895	0.035	0.032	0.036
Cu	−0.902	0.055	−0.085	0.039
Mn	0.162	−0.737	−0.063	0.131
Pb	0.258	0.24	0.719	−0.17
Cd	0.141	0.745	−0.111	0.141
Ni	−0.094	−0.292	0.762	0.183
As	−0.0002	0.012	0.02	0.964
Percentage of variance (%)	24.788	17.782	16.051	14.751
Percentage of accumulation (%)	24.788	42.570	58.621	73.372

**Table 5:** Results of the evaluation of the $E_{r}^{i}$ for heavy metals.

	$$E_{r}^{i}$$		
	Min	Max	Contribution (%)
Zn	0.24	8.08	1.86
Cu	3.06	19.55	8.78
Mn	0.01	1.56	0.17
Pb	0.15	38.96	5.37
Cd	14	249.77	61.89
Ni	4.92	13.82	8.27
As	6.15	23.69	13.66
RI	51.1	294.87	

## Data Availability

The data presented in this paper are available on request from the corresponding author.
